# Molecular and Clinical Screening of Selected Feline Viral Infections in Sulaymaniyah, Kurdistan Region, Iraq

**DOI:** 10.1155/av/5558740

**Published:** 2026-06-29

**Authors:** Sirwan Sleman, Basm Ali, Zaniar A. Abass, Barham J. Abdullah, Omed I. Abid, Masood B. Ameen, Rand Swara, Ali Sirwan

**Affiliations:** ^1^ College of Veterinary Medicine, University of Sulaimani, Sulaymaniyah, Iraq, univsul.edu.iq; ^2^ Sulaimani Veterinary Directorate, Sulaimani Veterinary Laboratory, Sulaymaniyah, Iraq

## Abstract

This study presents a new molecular epidemiology dataset on the prevalence of selected viral pathogens, including Feline panleukopenia virus (FPV), feline calicivirus (FCV), and feline coronavirus (FCoV) (systemic feline infectious peritonitis [FIP]), in domestic cats in Sulaymaniyah, Kurdistan region, Iraq. From October 2024 to January 2025, a total of 145 clinically suspected cats aged 2–24 months presented to private veterinary hospitals were analyzed using quantitative PCR (qPCR) and reverse transcription qPCR (RT‐qPCR) on blood, feces, oral swabs, and abdominal effusion. Our findings revealed that 86/145 (59.3%) cats were positive for at least one virus: FCoV (including FIP) was detected in 13/25 (52%) samples, FPV in 63/102 (61.8%), and FCV in 10/20 (50%). All categorical comparisons are presented with explicit numerical values (n and %), improving interpretability. Most positive cases were reported among younger cats (2–12 months). Additionally, infection prevalence was higher in nonvaccinated (25.9%) and stray cats (22.5%) compared with vaccinated cats (15.0%). Vaccination status was recorded; however, detailed vaccine type and schedule were not consistently available. These findings suggest a strong association between vaccination status and infection risk, underscoring the importance of immunization programs. This study establishes the first molecular baseline for feline viral infections in Sulaymaniyah and supports the development of targeted surveillance, vaccination strategies, and improved veterinary public health interventions in the region.

## 1. Introduction

Feline viral infections present considerable health threats to domestic and shelter cats globally, with significant consequences for animal welfare, public health, and veterinary resources. The prevalent viruses of interest—feline coronavirus (FCoV), feline panleukopenia virus (FPV), and feline calicivirus (FCV)—represent major etiological agents responsible for a wide spectrum of clinical manifestations worldwide [1, 2]. These infections range from mild upper respiratory disease to severe systemic illness and death, particularly in young, immunocompromised, or unvaccinated animals. Understanding the epidemiology, transmission dynamics, and risk factors associated with these infections is essential for developing effective prevention and control strategies [3, 4].

FCoV, a positive‐sense RNA virus of the *Coronaviridae* family, is widely distributed globally and infects cats of all ages, with the highest incidence reported in young cats around 6 months of age [5]. Seroprevalence in healthy populations ranges from 20% to over 80%, and may reach nearly 100% in high‐density or multicat environments, while remaining lower (10%–50%) in single‐cat households [5]. FCoV exists in two biotypes: feline enteric coronavirus (FECV), which typically causes mild or subclinical intestinal infection, and feline infectious peritonitis virus (FIPV), a mutated form capable of systemic dissemination and causing the often fatal disease feline infectious peritonitis (FIP) [6, 7]. The progression from FECV to FIPV is influenced by multiple factors, including host immune response, viral mutation, age, and environmental conditions [8, 9].

FPV, a DNA virus of the *Parvoviridae* family, is a highly contagious and frequently lethal pathogen affecting both domestic and wild felids worldwide. Infection leads to severe immunosuppression, gastrointestinal damage, and high mortality rates, particularly among kittens and unvaccinated animals [10–13]. Despite widespread vaccination efforts, FPV outbreaks continue to occur, especially in high‐density and poorly vaccinated populations [10, 11]. The virus is highly resistant and can persist in the environment for prolonged periods, complicating control measures [12]. Reported prevalence rates vary between 8.57% and 40.45%, with higher rates consistently observed in young and unvaccinated cats [13–15]. Mortality rates range from 25% to 90% and may approach 100% in severe acute infections [16].

FCV, a small single‐stranded RNA virus within the *Caliciviridae* family, is characterized by high genetic variability, resulting in the emergence of multiple viral strains with varying pathogenicity [1, 17, 18]. FCV is a major contributor to the feline upper respiratory disease complex and is frequently associated with coinfections involving other viral pathogens. Clinical manifestations include sneezing, nasal discharge, oral ulceration, and chronic carrier states, facilitating ongoing transmission within cat populations. Studies have reported high prevalence rates of FCV in clinically affected cats, often exceeding 50% in symptomatic populations [17, 18].

Importantly, coinfections involving FPV, FCV, and FCoV are increasingly recognized, particularly in high‐density environments such as shelters and stray populations, where they may exacerbate disease severity and complicate clinical diagnosis [3, 4].

Currently, there is a significant gap in the epidemiological understanding of these viral infections in Iraq, particularly in Sulaymaniyah. While studies from Baghdad, Mosul, and Duhok have reported the presence and molecular detection of these viruses [19–21], no comprehensive molecular survey has been conducted in Sulaymaniyah. Furthermore, regional comparisons with neighboring countries highlight similar epidemiological patterns, emphasizing the need for localized data to guide control strategies.

Accurate and early diagnosis of feline viral infections is critical, as these diseases often present with overlapping clinical signs. Molecular diagnostic tools such as polymerase chain reaction (PCR) and reverse transcription PCR (RT‐PCR) have become essential for detecting active infections with high sensitivity and specificity and play a crucial role in surveillance and disease management [22].

The population of pet and stray cats in Sulaymaniyah is increasing, yet there is limited information on the prevalence, transmission dynamics, and control of feline viral diseases in this region. In addition, variations in vaccination coverage and a lack of standardized vaccination records further complicate epidemiological assessments. To address this gap, the present study provides the first molecular investigation of FPV, FCV, and FCoV in domestic cats in Sulaymaniyah, integrating clinical and molecular data to support improved veterinary practices, public health planning, and disease control strategies.

## 2. Materials and Methods

### 2.1. Study Area

This study was conducted in Sulaymaniyah, Kurdistan Region, Iraq, between October 2024 and January 2025, using samples collected from private veterinary clinics in the city.

### 2.2. Study Population and Sample Collection

A total of 145 cats presenting with respiratory, oral, gastrointestinal, or systemic clinical signs were included in this study. The age of animals ranged from 2 to 24 months.

Samples were collected under aseptic conditions and included oral swabs, eye swabs, feces, blood, and abdominal fluid. Demographic data, including age, vaccination status (vaccinated, nonvaccinated, or stray), and clinical signs, were recorded for each animal.

Detailed vaccination schedules and vaccine types were not consistently available due to variability in clinical records.

### 2.3. Primers

Table 1 lists the primer pairs used to amplify FCoV, FPV, and FCV target genes. Primers targeting FPV (VP2 gene), FCoV, and FCV were selected based on previously validated studies to ensure assay specificity and sensitivity [23–25].

**TABLE 1:** lists PCR primer sequences for three feline viruses—feline panleukopenia virus (FPV), feline coronavirus (FCoV), and feline calicivirus (FCV)—along with their annealing temperatures, expected amplicon (PCR product) sizes, and literature references.

Virus	Primer (5′ ⟶ 3′)	Tm (°C)	Size (bp)	Reference
FPV	F^∗^‐ATTGATGGAGTCTTCTGGGTTGC	56	345 bp	[23]
	R^∗^‐TCGGGTGTTTCTCCTGTTGTAG			
FCoV	F‐CTACTCTTGTACAGAATGG	53	221 bp	[24]
	R‐TGTGTATCACTATCAAAAGG			
FCV	F‐GTTGACCCTTACTCATACAC	53	136 bp	[25]
	R‐CCCTGGGGTTAGGCGC			

*Note:* Each virus has a forward (F) and reverse (R) primer pair. The amplicon sizes are 345 bp for FPV (annealing at 56°C), 221 bp for FCoV (53°C), and 136 bp for FCV (53°C). These primers would be used in multiplex or individual PCR assays to detect and differentiate the three viruses based on product size.

^∗^F = forward, R = reverse.

### 2.4. Nucleic Acid Extraction

Viral nucleic acids were extracted from 100 to 200 μL of each sample using the AddPrep Viral DNA/RNA Kit (ADDBIO, Korea), following the manufacturer’s instructions.

### 2.5. Reverse Transcription (RT Step for RNA Viruses)

Reverse transcription was performed for RNA viruses (FCoV and FCV). A 15‐μL reaction mixture containing binding buffer, MgCl_2_, dNTPs, nuclease inhibitor, random hexamers, Multiscribe reverse transcriptase, and nuclease‐free water was added to RNA templates.

Cycling conditions were as follows: 30 min at 48°C, followed by enzyme inactivation at 95°C for 10 min.

### 2.6. Real‐Time PCR (Quantitative PCR [qPCR] and Reverse Transcription qPCR [RT‐qPCR])

qPCR was performed for FPV (DNA virus), while RT‐qPCR was used for RNA viruses (FCoV and FCV).

Each 20‐μL reaction contained 10‐μL SYBR Green master mix, 2.5‐μL forward primer (20 μM), 2.5‐μL reverse primer (20 μM), and 5‐μL template. Amplification was performed using a Coyote Mini 8 system under the following conditions:

• Initial denaturation: 95°C for 15 s
• Annealing/extension: 60°C for 60 s
• 40 amplification cycles

Melt curve analysis was performed at 95°C for 15 s, 60°C for 15 s, and 95°C for 15 s.

Positive results were defined by the following:

• A clear sigmoid amplification curve (Ct value within detectable range), and
• A virus‐specific melting temperature (Tm):
  - FCoV (FIP): 70°C–78°C
  - FPV: 75°C–80°C
  - FCV: 80°C–84°C

Both Ct values and melt peak concordance were required to define true‐positive results, improving diagnostic specificity and reducing false positives. Details of positive control materials and kits are provided in Supporting Table S1.

## 3. Results

### 3.1. Validation and Controls

Positive control plasmids and no‐template (negative) controls were included in each run.

Positive controls included plasmid DNA targeting FPV (VP2), FCV (ORF1), and FCoV sequences, ensuring assay validation and reproducibility.

**FIGURE 1:**
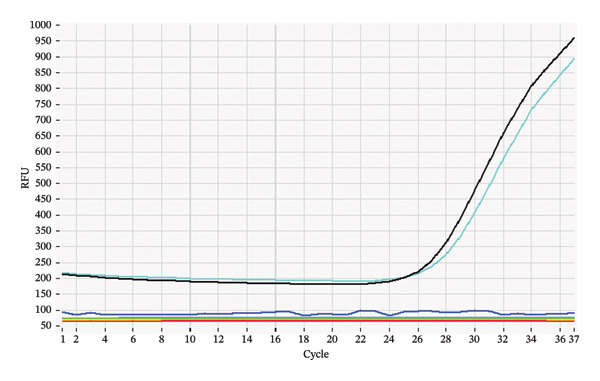
The amplification plot for a PCR run. It shows Ct values for the extracted FIP virus (FCoV) sample positive (light green) and negative (dark green), positive control (black), and negative controls (red) at a cycling condition of 60°C. The data shown here represent a single experiment.

**FIGURE 2:**
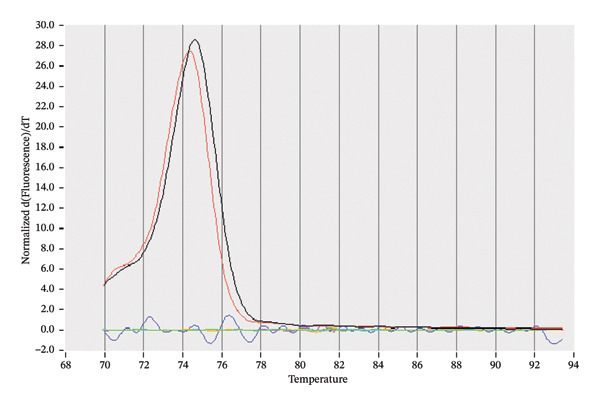
Derivative melting curve for a PCR run. It shows specific melting temperature (Tm) for extracted FIP virus (FCoV) positive (red), negative extract (No template ^∗^) (blue), positive control template (black), and negative PCR controls (No template) (green) at a cycling condition of 60°C. The data shown here represent a single experiment. ^∗^Nuclease‐free water.

Amplification plots and melting curve analyses were used to confirm specificity (Figures 1 and 2 and Supporting Figures 3–6). These validation steps ensured reliability in the absence of sequencing data.

### 3.2. Detection of Systemic FCoV (FIP Cases)

In several cases, FCoV was detected by RT‐qPCR from blood, ascitic fluid, or eye discharge, indicating systemic infection consistent with FIP.

Ct values demonstrated detectable viral loads within expected ranges for disseminated infection, and melting curve analysis confirmed specificity (Tm 70°C–78°C).

### 3.3. Data Handling and Analysis

Animal data, including age, vaccination status, clinical signs, and laboratory results, were recorded (Supporting Table S2).

Prevalence was calculated as (positive/tested × 100), and results were reported with explicit numerical values (n and %).

Associations between infection, age group, and vaccination status were analyzed descriptively to identify epidemiological trends.

A total of 86/145 (59.3%) cats tested positive for at least one target virus. The distribution of infection according to vaccination status is presented in Supporting Table S3.

FPV was predominantly detected in fecal samples, showing the highest positivity rate (63/102; 61.8%). FCV was identified in 50% of the tested oral swabs (10/20), confirming its strong association with oral and upper respiratory manifestations.

FCoV, associated with FIP, was detected in both blood and abdominal fluid samples, with a higher positivity rate observed in blood (11/13; 84.6%) compared with abdominal fluid (2/10; 20.0%). These findings indicate systemic involvement of FCoV in clinically suspected cases rather than lower overall prevalence. Table 2 summarizes virus detection by sample type, including the number of tested and positive samples and the corresponding prevalence for each virus.

**TABLE 2:** Virus detection by sample type.

Virus	Sample type	Tested (*n*)	Positive (*n*)	Prevalence (%)
FPV	Feces	102	63	61.8
FCoV (FIP)	Blood	13	11	84.6
FCoV (FIP)	Abdominal fluid	10	2	20.0
FCV	Oral swab	20	10	50.0

Virus‐specific prevalence according to vaccination status is summarized in Supporting Table S4. FPV, FCV, and FCoV were consistently detected more frequently among nonvaccinated and stray cats than among vaccinated animals.

## 4. Discussion

This study provides the first molecular evidence of FCoV, FPV, and FCV circulation in domestic cats in Sulaymaniyah, Kurdistan Region, Iraq, based on validated qPCR and RT‐qPCR assays. The integration of clinical data with molecular diagnostics strengthens the epidemiological and translational relevance of these findings.

FPV was the most prevalent virus detected (63/102; 61.8%), highlighting its dominant role in feline infectious disease within the study population. This high prevalence is consistent with the known environmental stability and high transmissibility of FPV, particularly in young and unvaccinated cats [10–13]. FCV was detected in 50% of oral swab samples from cats presenting with respiratory and oral lesions, supporting its well‐established role in feline upper respiratory disease complexes [17, 18]. FCoV (including FIP‐associated cases) was detected in 52% of the tested blood and effusion samples, reflecting systemic involvement in clinically suspected cases.

The high overall positivity rate (86/145; 59.3%) likely reflects targeted sampling of clinically affected animals rather than the general population and should be interpreted within this clinical context.

In agreement with previous studies, the majority of positive cases occurred in young cats (2–12 months), confirming age as a major risk factor for infection [13]. This age‐related susceptibility is likely linked to immature immune responses and increased exposure in multicat or high‐density environments. A summary of major epidemiological risk factors identified in this study is provided in Supporting Table S5.

The study further demonstrates a clear association between infection prevalence and vaccination status. The overall prevalence of viral infections was higher in nonvaccinated (25.9%) and stray cats (22.5%) compared to vaccinated animals (15.0%). Similarly, virus‐specific prevalence rates were consistently higher in nonvaccinated and stray groups. These findings strongly support the protective role of vaccination in reducing infection risk. However, interpretation should consider that detailed vaccination schedules and vaccine strain information were not consistently available.

In addition, the observed infection patterns suggest the possible contribution of coinfections and environmental exposure in high‐density or unmanaged populations, which may exacerbate disease severity and complicate clinical outcomes.

When compared with regional studies, our findings are consistent with a broader pattern of high viral circulation among feline populations in Iraq and neighboring countries. The FPV prevalence (61.8%) is comparable to a study from Duhok reporting approximately 70% PCR positivity, particularly among young and stray cats [21]. Similarly, the FCV detection rate (∼50%) falls within the upper range reported in symptomatic cats (23%–50%) in regional and international studies [26].

FCoV detection in this study is also comparable to findings from Iraq and nearby regions, where PCR positivity rates around 20% are reported in clinically suspected FIP cases [27]. This supports the concept that while FCoV is widely circulating, only a subset of infections progress to clinical FIP, likely influenced by host and environmental factors [6–9].

The use of sensitive molecular techniques (qPCR and RT‐qPCR), combined with strict validation criteria (Ct values and melting curve analysis), likely contributed to the relatively high detection rates observed in this study.

Collectively, these findings suggest that the high prevalence observed is driven by a combination of factors, including targeted sampling of symptomatic animals, increased susceptibility in young and unvaccinated populations, environmental persistence of viruses such as FPV, and gaps in vaccination coverage.

## 5. Limitations and Considerations

• The study population consisted of clinically presented cases from private hospitals, which may not fully represent the general cat population.
• Lack of sequencing data limited the ability to characterize circulating viral strains and assess vaccine–strain relationships.
• Incomplete vaccination records restricted detailed analysis of vaccine efficacy and immunization schedules.
• Clinical and public health implications

Routine molecular diagnostics (qPCR/RT‐qPCR) can significantly improve diagnostic accuracy, clinical decision‐making, and prognosis in veterinary practice.

The findings highlight the urgent need for structured vaccination programs, particularly targeting kittens and stray populations, as well as improved public awareness regarding feline health and disease prevention.

## 6. Future Directions

Future studies should incorporate genomic sequencing, longitudinal surveillance, and risk factor analysis to better understand viral evolution, transmission dynamics, and vaccine effectiveness in the region.

## Author Contributions

Sirwan Sleman conceived and supervised the study, coordinated data collection, analyzed and interpreted the data, and drafted and revised the manuscript. Zaniar A. Abass, Barham J. Abdullah, Basm Ali, Omed I. Abid, and Masood B. Ameen contributed to writing, editing, and reviewing, data acquisition, interpretation of findings, and critical revision of the manuscript.

## Funding

This research received no specific grant from any funding agency in the public, commercial, or not‐for‐profit sectors.

## Disclosure

All authors have reviewed and approved the final manuscript and agreed to be accountable for all aspects of the work.

## Ethics Statement

This study was based exclusively on the laboratory analysis of clinical diagnostic specimens that had already been collected by licensed veterinarians and clinical staff as part of routine veterinary diagnostic procedures. The authors did not directly collect samples from animals, perform animal experiments, or conduct any interventions involving live animals for research purposes. Accordingly, the study represents a retrospective analysis of pre‐existing diagnostic specimens and anonymized clinical records. Formal animal experimentation ethics approval was, therefore, not considered applicable to the study design. Where required, permission for the use of anonymized diagnostic data and specimens for research purposes was obtained from the participating veterinary clinics and hospitals.

## Consent

All specimens included in this study were obtained during routine veterinary diagnostic investigations conducted by the attending veterinarians. Clinical information was anonymized before analysis, and no personally identifiable information relating to animal owners was collected or reported.

## Conflicts of Interest

The authors declare no conflicts of interest.

## Supporting Information

Additional supporting information can be found online in the Supporting Information section.

## Supporting information


**Supporting Information** Table S1. Positive controls and validation materials. This table shows the positive control materials used to validate qPCR and RT‐qPCR assays for the detection of FCoV, FCV, and FPV, ensuring assay specificity and reliability. Table S2. Clinical and molecular data of representative cases. This table presents the clinical findings, vaccination status, sample types, and molecular diagnostic results of 30 representative cats tested for major feline viral infections. Positive cases included feline calicivirus (FCV), feline panleukopenia virus (FPV), and feline coronavirus (FCoV/FIP), while some clinically suspected cases tested negative. Ct values ranged from 15–29 among positive samples. Table S3. Overall prevalence of viral infections by vaccination status. This table illustrates the prevalence of all virus infections in vaccinated, nonvaccinated, and stray cat groups. *N* = number of unique Animal_IDs that have that virus mentioned (in any row), Infected = number of Animal_IDs with at least one Positive result for that virus, and Prevalence = (Infected / *N*) × 100, shown to 2 decimals. Table S4. Virus‐specific prevalence by vaccination status. This table shows the prevalence of FCV, FPV, and FCoV separately in vaccinated, nonvaccinated, and stray cat groups. N = number of unique Animal_IDs that have that virus mentioned (in any row), Infected = number of Animal_IDs with at least one positive result for that virus, and Prevalence = (Infected / N) × 100, shown to 2 decimals. Table S5. Risk factor analysis. This table shows that younger cats (≤ 6 months) and nonvaccinated cats had higher infection rates, suggesting that age and vaccination status are important risk factors for feline viral infections. Figure S3. Amplification plot for FPV virus. The amplification plot for a PCR run shows Ct values for the extracted FPV virus sample positive (Black) and negative (blue), at a cycling condition of 60°C. The data shown here represent a positive sample in a single experiment. Figure S4. Derivative melting curve for FPV virus Derivative melting curve for a PCR run shows specific melting temperature (Tm) for extracted FPV virus positive (BLACK) and negative (RED), at a cycling condition of 60°C. The data shown here represent a positive sample in a single experiment. Figure S5. Amplification plot for FCV virus The amplification plot for a PCR run shows Ct values for the extracted FCV sample positive (blue) and negative (Red), at a cycling condition of 60°C. The data shown here represent a positive sample in a single experiment. Figure S6. Derivative melting curve for FCV virus The derivative melting curve for a PCR run shows a specific melting temperature (Tm) for extracted FCV positive (blue) and negative (Red), at a cycling condition of 60°C. The data shown here represent a single experiment.

## Data Availability

The data supporting the findings of this study were derived from routine diagnostic submissions and anonymized clinical records provided by participating veterinary clinics and hospitals. Owing to institutional confidentiality policies and client privacy considerations, the complete dataset is not publicly available. Relevant data may be made available from the corresponding author upon reasonable request and with permission from the participating institutions.
