# Supplementary material for: Molecular and Clinical Screening of Selected Feline Viral Infections in Sulaymaniyah, Kurdistan Region, Iraq
Source: Adv Virol. 2026 Jun 29;2026:5558740. doi: 10.1155/av/5558740 (PMC13312980; doi:10.1155/av/5558740)
Supplement: Supplementary file 1 — Supporting Information Table S1. Positive controls and validation materials. This table shows the positive control materials used to validate qPCR and RT‐qPCR assays for the detection of FCoV, FCV, and FPV, ensuring assay specificity and reliability. Table S2. Clinical and molecular data of representative cases. This table presents the clinical findings, vaccination status, sample types, and molecular diagnostic results of 30 representative cats tested for major feline viral infections. Positive cases included feline calicivirus (FCV), feline panleukopenia virus (FPV), and feline coronavirus (FCoV/FIP), while some clinically suspected cases tested negative. Ct values ranged from 15–29 among positive samples. Table S3. Overall prevalence of viral infections by vaccination status. This table illustrates the prevalence of all virus infections in vaccinated, nonvaccinated, and stray cat groups. N = number of unique Animal_IDs that have that virus mentioned (in any row), Infected = number of Animal_IDs with at least one Positive result for that virus, and Prevalence = (Infected / N) × 100, shown to 2 decimals. Table S4. Virus‐specific prevalence by vaccination status. This table shows the prevalence of FCV, FPV, and FCoV separately in vaccinated, nonvaccinated, and stray cat groups. N = number of unique Animal_IDs that have that virus mentioned (in any row), Infected = number of Animal_IDs with at least one positive result for that virus, and Prevalence = (Infected / N) × 100, shown to 2 decimals. Table S5. Risk factor analysis. This table shows that younger cats (≤ 6 months) and nonvaccinated cats had higher infection rates, suggesting that age and vaccination status are important risk factors for feline viral infections. Figure S3. Amplification plot for FPV virus. The amplification plot for a PCR run shows Ct values for the extracted FPV virus sample positive (Black) and negative (blue), at a cycling condition of 60°C. The data shown here represent a positive sample i [file AV-2026-5558740-s001.docx]

**Supplementary Tables and Figures**

**Molecular and Clinical Screening of Selected Feline Viral Infections in Sulaymaniyah, Kurdistan Region, Iraq**

Sirwan Sleman^1,*^, Basm Ali^1^ , Zaniar A. Abass^2^, Barham J. Abdullah^2^, Omed I. Abid^2^ , Masood B. Ameen^2^, Rand Swara^2^, Ali Sirwan^2^

¹ College of Veterinary Medicine, University of Sulaimani, Sulaymaniyah, Iraq.

^2^ Sulaimani Veterinary Directorate, Sulaimani Veterinary Laboratory, Sulaymaniyah, Iraq

***Corresponding** author: Assist. Prof. Dr Sirwan Sleman

**E-mail:** sirwan.sleman@univsul.edu.iq

**Tel**: +964-772-152-5583

**Running title:** Most prevalent cat viruses

**Supplementary Tables:**

**Table S1. Positive Controls and Validation Materials**.

| Virus | Target Gene | Detection Method | Positive Control Material | Source |
| --- | --- | --- | --- | --- |
| FCoV | Conserved region | RT-qPCR | Plasmid DNA (pEX-A) containing the coronavirus sequence | [24,26] |
| FCV | orf1 gene | RT-qPCR | Plasmid DNA containing the FCV ORF1 fragment | [25] |
| FPV | VP2 gene | qPCR | Plasmid DNA containing the VP2 gene | [23] |

**Table S2.** **Clinical and Molecular Data of Representative Cases**.

| Animal ID | Age (months) | Vaccination Status | Clinical Signs | Sample Type | | Detected Virus | Ct Value | | Result | |
| --- | --- | --- | --- | --- | --- | --- | --- | --- | --- | --- |
| A-001 | 7 | Not vaccinated | Sneezing, nasal discharge, tongue ulceration | | Oral swab | | FCV | 19 | | Positive |
| A-001 | 7 | Not vaccinated | Eye discharge | | Eye swab | | FCV | 21 | | Positive |
| A-002 | 12 | Stray | Vomiting, diarrhoea, fever, seizure | | Faeces | | FPV | 16 | | Positive |
| A-003 | 4 | Stray | Diarrhoea, vomiting | | Faeces | | FPV | 17 | | Positive |
| A-004 | 2.5 | Not vaccinated | Vomiting, weakness | | Faeces | | FPV | 18 | | Positive |
| A-005 | 7 | Not vaccinated | Tongue ulceration | | Oral swab | | FCV | 21 | | Positive |
| A-006 | 7 | Not vaccinated | Gingivitis, oral ulcer, vomiting | | Oral swab | | None | – | | Negative |
| A-007 | 7 | Stray | Lethargy, hypothermia, dental plaque | | Oral swab | | None | – | | Negative |
| A-008 | 24 | Vaccinated | Vomiting, dehydration, oral ulceration | | Oral swab | | FCV | 22 | | Positive |
| A-009 | 8 | Not vaccinated | Fever, vomiting, oral lesion | | Oral swab | | None | – | | Negative |
| A-010 | 3 | Not vaccinated | Anorexia, fever, tongue ulceration | | Oral swab | | FCV | 20 | | Positive |
| A-011 | 4 | Stray | Lethargy, diarrhea, gingivitis | | Oral + Faeces | | None | – | | Negative |
| A-012 | 14 | Not vaccinated | Lethargy, diarrhea | | Faeces | | None | – | | Negative |
| A-013 | 12 | Vaccinated | Vomiting, diarrhea, anorexia | | Faeces | | None | – | | Negative |
| A-014 | 5 | Stray | Tongue ulceration, lethargy | | Oral swab | | FCV | 16 | | Positive |
| A-015 | 4 | Vaccinated | Ascites, lethargy, jaundice | | Blood | | FCoV (FIP) | 17 | | Positive |
| A-016 | 6 | Not vaccinated | Dyspnea, ascites | | Blood | | FCoV (FIP) | 23 | | Positive |
| A-017 | 4 | Stray | Ascites | | Blood | | FCoV (FIP) | 20 | | Positive |
| A-018 | 5 | Not vaccinated | Dyspnea, ascites | | Blood | | FCoV (FIP) | 24 | | Positive |
| A-019 | 2 | Vaccinated | Diarrhoea, lethargy, ascites | | Faeces | | FPV | 22 | | Positive |
| A-020 | 3 | Stray | Ascites | | Blood | | FCoV (FIP) | 17 | | Positive |
| A-021 | 4 | Not vaccinated | Diarrhea | | Faeces | | FPV | 19 | | Positive |
| A-022 | 3 | Not vaccinated | Ascites | | Blood | | None | – | | Negative |
| A-023 | 3 | Not vaccinated | Lethargy, nasal discharge, ascites | | Blood | | FCoV (FIP) | 16 | | Positive |
| A-024 | 5 | Stray | Ascites | | Blood | | FCoV (FIP) | 15 | | Positive |
| A-025 | 7 | Not vaccinated | Tongue ulceration, anorexia | | Oral swab | | None | – | | Negative |
| A-026 | 3 | Not vaccinated | Bloody diarrhoea, vomiting | | Faeces | | None | – | | Negative |
| A-027 | 4 | Vaccinated | Ascites | | Blood | | FCoV (FIP) | 23 | | Positive |
| A-028 | 3 | Not vaccinated | Dyspnea, ascites | | Blood | | None | – | | Negative |
| A-029 | 4 | Not vaccinated | Ascites | | Blood | | FCoV (FIP) | 29 | | Positive |
| A-030 | 9 | Not vaccinated | Respiratory distress, jaundice | | Blood | | FCoV (FIP) | 22 | | Positive |

**Table S3. Overall prevalence of viral infections by vaccination status.**

| Vaccination Status | N (Total Cats) | Infected (n) | Prevalence (%) |
| --- | --- | --- | --- |
| Vaccinated | 40 | 6 | 15.00 |
| Not Vaccinated | 65 | 17 | 26.15 |
| Stray | 40 | 9 | 22.50 |
| Total | **145** | **86** | **59.31** |

**Table S4. Virus-Specific Prevalence by Vaccination Status.**

| Virus | Vaccination Status | Tested (n) | Positive (n) | Prevalence (%) |
| --- | --- | --- | --- | --- |
| FPV | Vaccinated | 40 | 2 | 5.0% |
| FPV | Not vaccinated | 40 | 12 | 30.0% |
| FPV | Stray | 20 | 6 | 30.0% |
| FCV | Vaccinated | 40 | 6 | 15.0% |
| FCV | Not vaccinated | 40 | 8 | 20.0% |
| FCV | Stray | 20 | 5 | 25.0% |
| FCoV | Vaccinated | 40 | 2 | 5.0% |
| FCoV | Not vaccinated | 40 | 8 | 20.0% |
| FCoV | Stray | 20 | 5 | 25.0% |

**Table S5. Risk Factor Analysis**.

| Variable | Category | Infection Rate (%) |
| --- | --- | --- |
| Age | ≤6 months | High |
| Age | >6 months | Lower |
| Vaccination | Vaccinated | 15% |
| Vaccination | Non-vaccinated | 26% |
| Status | Stray | 22.5% |

**Supplementary Figures:**


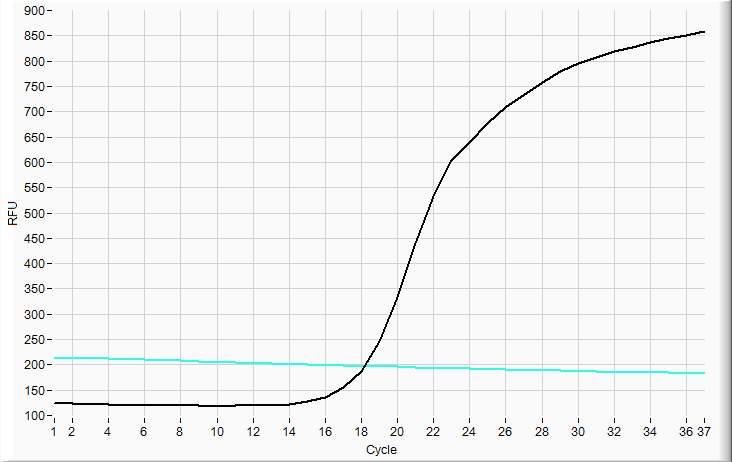


**Figure S3.** **Amplification plot for FPV virus**


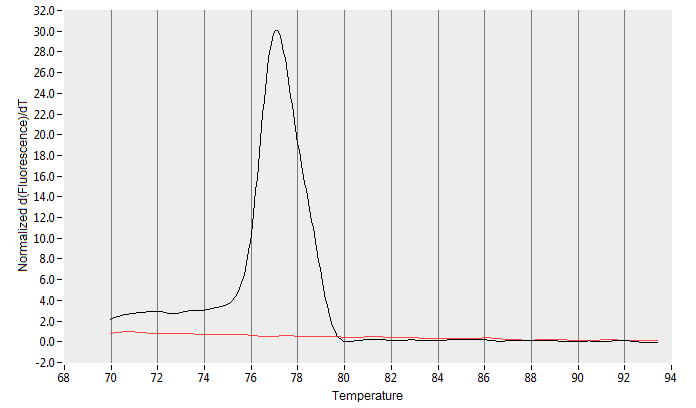


**Figure S4.** **Derivative melting curve for FPV virus**


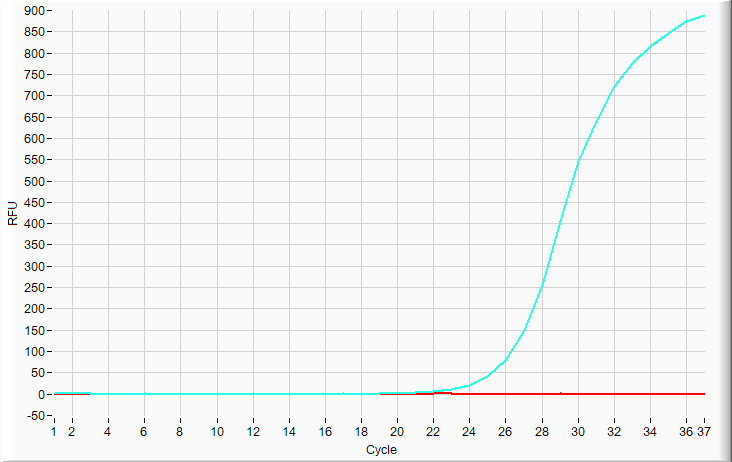


**Figure S5.** **Amplification plot for FCV virus**

**
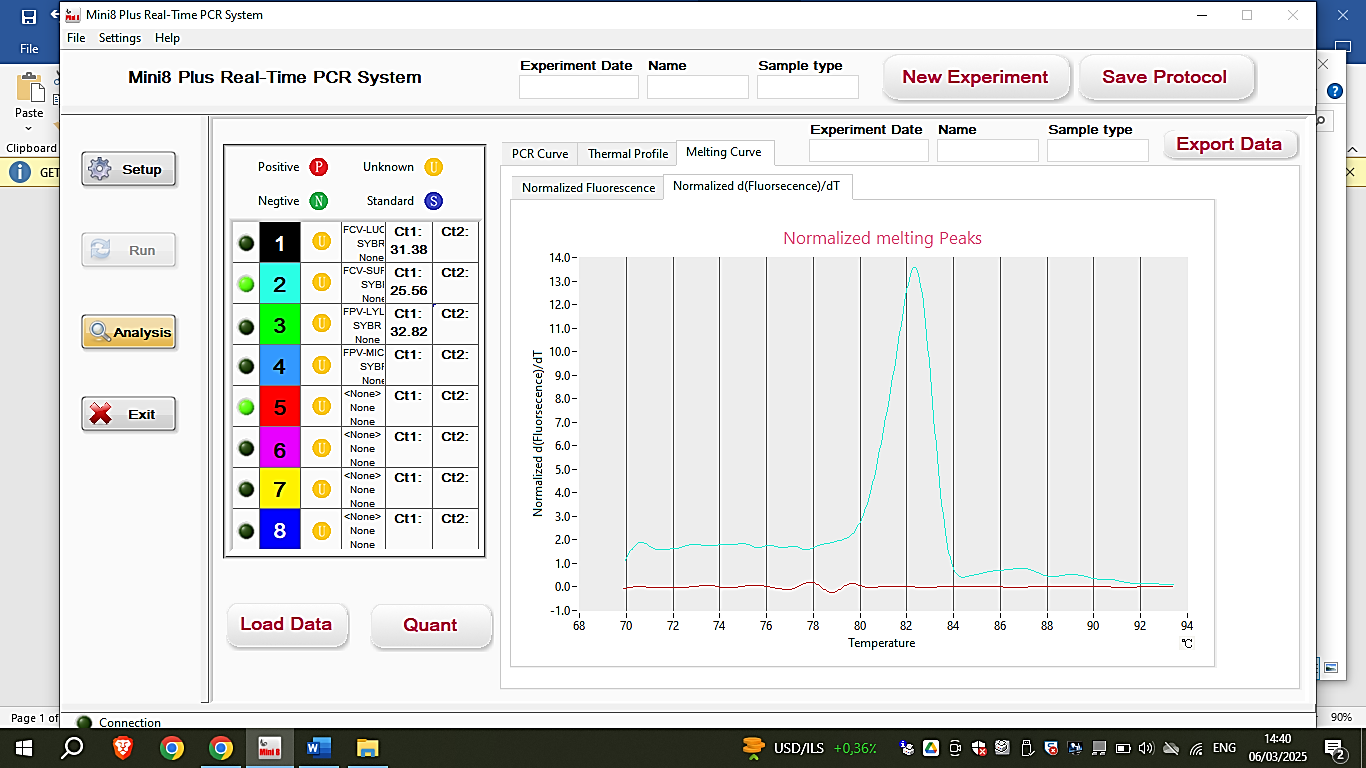
**

**Figure S6.** **Derivative melting curve for FCV virus**
